# Gas Chromatography-Mass spectrometry (GC-MS) Revealed Bioactive Constituents of *Brassica oleracea *var. *viridis* (Collard Greens) Used to Treat Male Infertility in Uganda.

**DOI:** 10.12688/f1000research.164618.2

**Published:** 2025-09-24

**Authors:** Emmanuel Orire Ikuomola, Daniel Udofia Owu, Victor Otu Oka, Sunday Agba Bisong, Ugwu Okechukwu Paul-Chima, Patrick Maduabuchi Aja

**Affiliations:** 1 Physiology, Faculty of Biomedical Sciences,, Kampala International University - Western Campus, Bushenyi, Western Region, 20000, Uganda; 2 Department of Physiology, University of Calabar, College of Medical Sciences, Calabar, Cross River, Nigeria; 3 Publication and Extension, Kampala International University - Western Campus, Bushenyi, Western Region, 20000, Uganda; 4 Biochemistry, Faculty of Biomedical Sciences, Kampala International University - Western Campus, Bushenyi, Western Region, 20000, Uganda

**Keywords:** Collard greens, GC-MS, Sukuma wiki, Phytochemicals, Phytol

## Abstract

**Background:**

Medicinal plants play a crucial role in pharmacological research, as many pharmaceutical companies rely on them for raw materials. Collard greens, also known as “Sukuma wiki,” are a member of the *Brassica oleracea* var. viridis family and are known for their medicinal properties. The aim of this study was to identify phytochemicals in the ethanol extract of collard green leaves that has traditional use in treating male infertility in Uganda.

**Method:**

The leaves were dried and crushed into fine powder and the powdered leaves (800 g) A was cold macerated in 5 liters of 99% ethanol (1:5 w/v) for 72 hours in sterile glass container. The ethanol extract (5g) was subjected to Gas Chromatography-Mass Spectrometry (GC-MS) analysis.

**Results:**

Phytochemical analysis of the Ethanol extract from *Brassica oleracea* var. viridis (Collard green) leaves revealed the presence of various compounds, including flavonoids, alkaloids, phenolic compounds, fatty acids, and terpenoids. Gas Chromatography-Mass Spectrometry (GC-MS) analysis identified 77 bioactive compounds, such as 2-Methoxy-4-vinylphenol, 2, 7-Dimethyl-1, 7-octadien-3-amine, Octadecanoic acid, 9, 12, 15-Octadecatrienoic acid, Pentadecanoic acid, and several phenolic derivatives.

**Conclusion:**

In conclusion, the phytochemicals identified in *Brassica oleracea* var. viridis (Collard greens), including Phytol, Omega-3 fatty acids, phenols, flavonoids, and sterols, demonstrate potential benefits for enhancing male fertility and reproductive health. These phytochemicals exert their beneficial action through their antioxidant, anti-inflammatory, and possibly neuroprotective properties. There is need for further research to establish the effectiveness of these phytochemicals in clinical settings and provide concrete guidelines for their use in treating male infertility.

**List of abbreviations**
DHA: Docosahexaenoic acid
GCMS: Gas Chromatography-Mass Spectrometry
IOE: Ikuomola Orire Emmanuel

## Introduction

For centuries, medicinal plants have played a vital role in traditional healing systems across cultures, primarily due to their rich content of bioactive compounds. ^1^ These phytochemicals such as flavonoids, alkaloids, tannins, terpenoids, and saponins have demonstrated significant therapeutic potential. ^2^ Acting individually or synergistically, they exhibit a broad spectrum of biological activities including antioxidant, anti-inflammatory, anti-diabetic, anti-cancer, and reproductive health-enhancing effects. ^3^ Their contribution to drug discovery continues to be profound, as the identification of novel therapeutic agents often begins with the screening of natural products from diverse plant sources. ^4^

**Figure 1.**
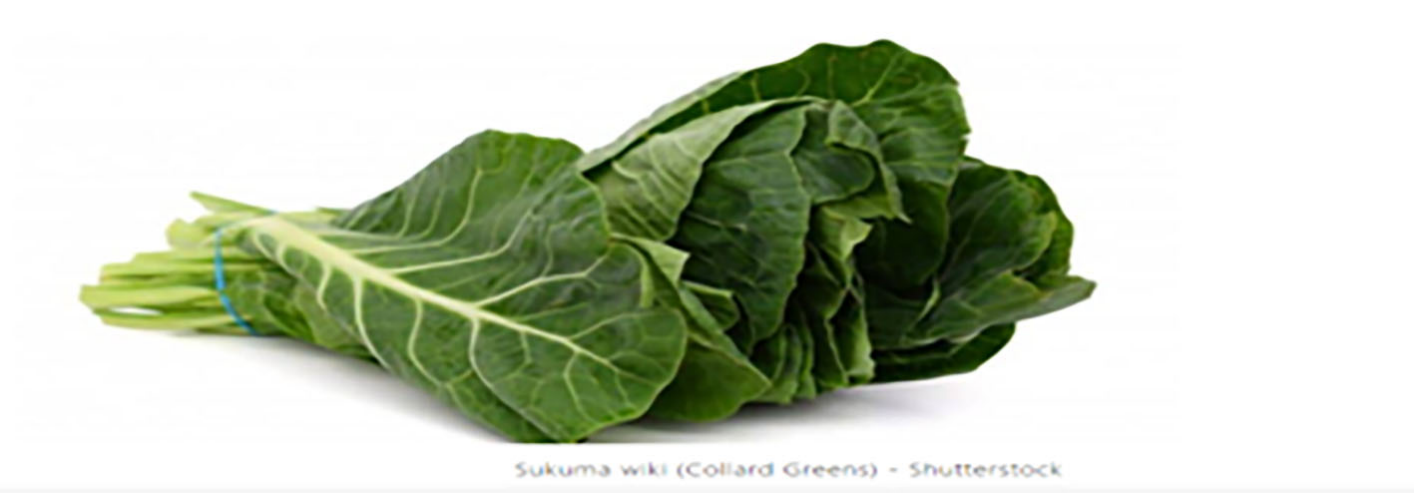
Diagram of *Brassica oleracea* var. viridis (collard green). ^20^

*Brassica oleracea* var. *viridis* (commonly known as collard greens or “Sukuma wiki” in Swahili) is a nutrient-rich leafy vegetable widely cultivated and consumed in Uganda and other parts of East Africa as shown in Figure 1. Characterized by large, broad leaves ranging in color from deep green to bluish-green, collard greens thrive in a variety of climates and are valued both as a food source and in ethnomedicine. Traditionally, this plant has been used to manage various health conditions, including male reproductive issues, though its phytochemical basis remains underexplored.

The health-promoting properties of collard greens are largely attributed to their high content of vitamins C, E, and K, dietary fiber, calcium, and a diverse range of bioactive compounds. These constituents support immune function, bone health, digestion, and oxidative balance. ^5^ Additionally, preliminary evidence suggests potential anti-hyperglycemic and fertility-enhancing effects, reinforcing its traditional use in managing male infertility. ^6, 7^

Gas Chromatography-Mass Spectrometry (GC-MS) is a robust and precise analytical technique employed in phytochemical investigations to identify and quantify chemical constituents in complex plant matrices. ^8, 9^ This method is especially effective in uncovering bioactive compounds that may explain the therapeutic value of traditionally used plants.

This study aims to analyze the bioactive constituents of *Brassica oleracea* var. *viridis* collected in Uganda using GC-MS. By identifying these compounds, the research seeks to provide scientific validation for the ethnomedicinal use of collard greens in the treatment of male infertility and contribute to the growing body of knowledge on plant-based fertility therapies.

## Methodology

### Chemicals

Only analytical-grade chemicals and reagents were used for the study.

### Plant collection and identification

Fresh leaves of *Brassica oleracea* var. *viridis* (collard greens) were collected from Bwejuragye in Ishaka Town, Bushenyi, Igara County, Western Uganda with GPS coordinates of approximately 0°32′42.0″S 30°08′18.0″Ephyt. The plant was identified and authenticated by Dr. Eunice Olet of the Department of Botany, Faculty of Biological Sciences, Mbarara University of Science and Technology. A voucher specimen was deposited in the university’s herbarium under the reference number IOE-24-001. Details of the plant collection and identification are presented in Table 1.

**Table 1.** Process of plant collection and identification.

PLANT COLLECTION DETAILS	
ITEMS	PROPERTIES
**DATE OF COLLECTION**	03-09-2024
**COLLECTION NUMBER**	IOE-24-001
**LOCAL NAME:**	Sukuma Wiki
**FAMILY NAME:**	Cruciferous Family/Acephala group
**GENERIC NAME:**	Chepkilumnda
**SCIENTIFIC NAME:**	*Brassica oleracea* var. viridis
**GROWTH FORM:**	Rosette Growth
**COLOR:**	Dark Green
**TYPE OF INFLORESCENCE:**	Raceme
**LEAF SHAPE:**	Ovate Shape
**TYPE OF MARGIN:**	Smooth or Slightly Undulating Margin
**LEAF VENATION:**	Palmate Venation Pattern
**PUBESCENCE:**	**Glabrous** (Smooth And Hairless) Leaves
**LEAF ARRANGEMENT:**	Alternate Leaf Arrangement
**COMMON PLANTS AROUND:**	Tomatoes, Onions, Garlic, Beans, Carrots
**TYPE OF ECO SYSTEM:**	Subtropical And Tropical Gardens
**LOCALITY:**	Bwejuragye, ishaka town
**HABITAT:**	Subtropical And Tropical Areas

### Preparation of plant material

Fresh leaves (2 kg) of *Brassica oleracea* var. *viridis* (collard greens) were collected from fields located in Bwejuragye, Bushenyi Local Government Area, Uganda. The samples were subsequently transported to the herbarium unit at Mbarara University for proper botanical identification and authentication. The leaves were thoroughly washed with distilled water to remove surface contaminants such as dirt and sand, and then shed-dried at ambient temperature of 26°C. After complete drying, the leaves were finely ground into a powder and stored in airtight containers in a dry environment for future extraction.

The extraction process was performed using a modified version of the method described by Abdulrahman et al. ^10^ A total of 800 g of the powdered leaves was cold macerated in 5 liter of 99% ethanol (1:5 w/v) for 72 hours in sterile glass container. The mixture was kept in the dark to prevent degradation at room temperature (26 **±** 2°C) with intermittent shaking every 6-12 hours to enhance solute diffusion and extraction efficiency. The resulting ethanolic mixture was then filtered through sterile Whatman No. 1 filter paper. The filtrate was concentrated to dryness reduced pressure using a rotary evaporator below 40°C having a constant weight. The dried crude extracts were stored at 4°C in an amber glass bottle that was tightly sealed until further analysis. Approximately 5 g of the ethanol extract was subjected to Gas Chromatography-Mass Spectrometry (GC-MS) analysis, and the remaining extract was stored in a refrigerator for subsequent use in further analyses.

### Gas Chromatography-Mass Spectrometry (GC-MS) analysis

GC-MS analysis was performed using a Shimadzu gas chromatograph-mass spectrometer. ^11, 12^ Chromatographic separation was achieved with a DB-5 fused-silica capillary column in inert MSD mode, equipped with a Triple-Axis detector. High-purity helium (99.999%) served as the carrier gas at a constant flow rate of 1.0 mL/min. The system was operated under the following conditions: ion source temperature at 250 °C, interface temperature at 300 °C, pressure at 16.2 psi, and out time of 1.8 mm. A 1 μL sample was injected in split mode (1:50) at 300 °C.

The column temperature program began at 36 °C (held for 5 min), ramped to 150 °C at 4 °C/min, then to 250 °C at 20 °C/min, and was held for 5 min, resulting in a total run time of 47.5 minutes. Relative percentage abundances were determined by comparing individual peak areas to the total chromatogram area. Data acquisition and instrument control were managed using MS Solution software, and compound identification was based on mass spectral comparisons with the NIST20 library.

## Results

Phytochemical screening of the Ethanol extraction of *Brassica oleracea* var. viridis (Collard Greens) leaves revealed the presence of the phytochemical constituent’s flavonoids, alkaloids, phenolic compounds, fatty acids, terpenoids, and other compounds as detailed in Table 2.

**Table 2.** Compounds present selected ethanol extract of *Brassica oleracea* var. viridis (collard greens) and their phytochemical properties.

S/N	Compound name	Phytochemical constituent
1	1-(Cyclopropylcarbonyl)-3-piperidinamine, N-trimethyl acetyl-	Alkaloids
2	6-Isobutyryl-2,2,4,4-tetramethylcyclohexane-1,3,5-trione	Terpenoids
3	Phenol, 4-ethenyl-2,6-dimethoxy-	Phenolics
4	4. 2,2,4,4-Tetramethyl-6-(2-methylbutanol) cyclohexane-1,3,5-trione	Terpenoids
5	Bacteriochlorophyll-c-stearyl	Chlorophylls
6	Dihydroisoobtusilactone	Terpenoids
7	7,10,13-Hexadecatrienoic acid, (Z, Z, Z)-	Fatty Acids
8	Pentadecanoic acid	Fatty Acids
9	Phytol	Terpenoids
10	9,12,15-Octadecatrienoic acid, (Z, Z,Z)-	Fatty Acids
11	Dichloroacetic acid, tridec-2-vinyl ester	Organic Acids
12	9,12,15-Octadecatrienoic acid, 2,3-hydroxypropyl ester, (Z,Z,Z)	Fatty acids
13	Hexatriacontane	Alkanes
14	Gamma-Sitosterol	Steriods

### Gas Chromatography-Mass Spectrometry (GC-MS) profiling of ethanol extract of Brassica oleracea var. viridis (Collard Greens)

**Figure 2.**
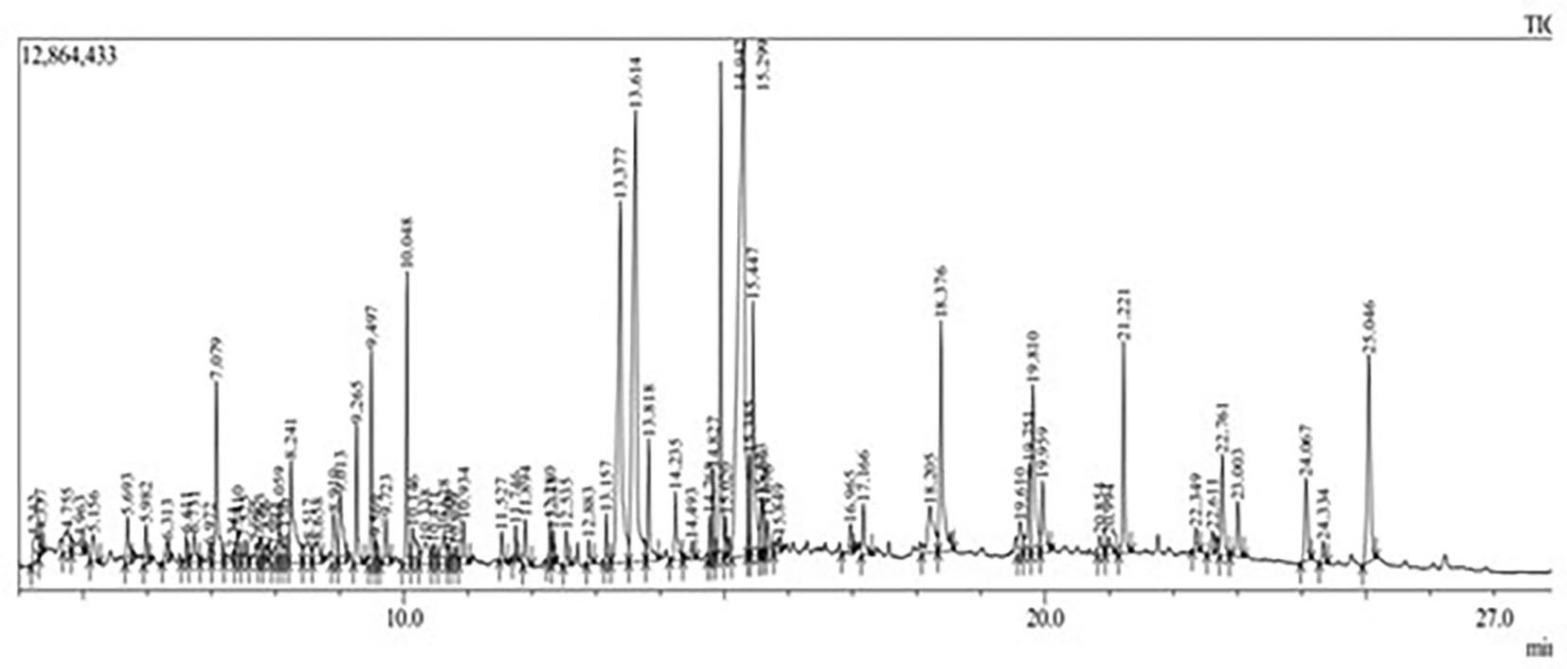
Showing the chromatograph of ethanol extract of Brassica oleracea var. viridis.

Gas Chromatography-Mass Spectrometry (GC-MS) analysis of the ethanol extract obtained from the leaves of *Brassica oleracea* var. *viridis* (collard greens) identified a total of 77 distinct phytochemical constituents exhibiting a broad spectrum of bioactive properties. The resulting chromatogram is presented in Figure 2, while Table 3 details the identified compounds, including those meeting the inclusion threshold of ≥1.90% area percentage. Compounds with an area percentage below this threshold were excluded from further analysis. The table also provides information on each compound’s retention time (RT), molecular formula, molecular weight (MW), and chemical structure.

**Table 3.** Selected by %age area (1.90 upward for inclusion criteria, exclusion is 1.90 downwards) of ethanol extract of *Brassica Oleracea* var viridis (collard greens).

S/N	Name	MF	MW	RT	Area%	Structure of compounds
1	1-(cyclopropyl carbonyl) piperidine-3-amine	C _9_H _16_N _2_O	168.24g/mol	9.021	2.33	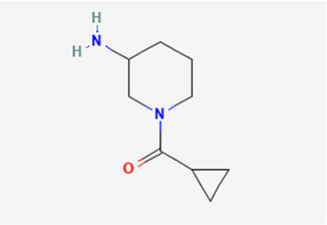
2	6-Isobutyryl-2,2,4,4-tetra methylcyclohexane-1,3,5-trione	C _14_H _20_O _4_	252.31g/mol	9.280	3.68	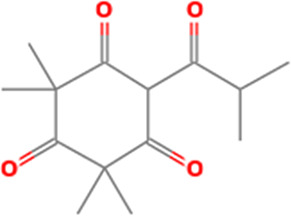
3	Phenol, 4-ethenyl-2,6-dimethoxy-	C _10_H _12_O _3_	180.2g/mol	9.508	1.90	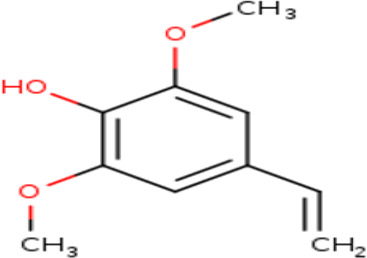
4	2,2,4,4-Tetramethyl-6-(2-methyl butanol) cyclohexane-1,3,5-trione	C _15_H _22_O _4_	266.33g/mol	10.080	5.90	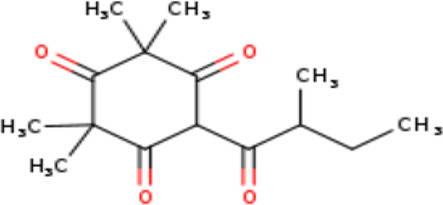
5	Bacteriochlorophyll-c-stearyl	C _52_H _72_MgN _4_O _4_^−2^	841.5g/mol	10.170	3.71	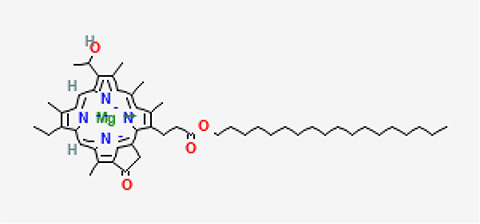
6	Dihydroisoobtusilactone	C _17_H _28_O _3_	280.408g/mol	10.943	3.23	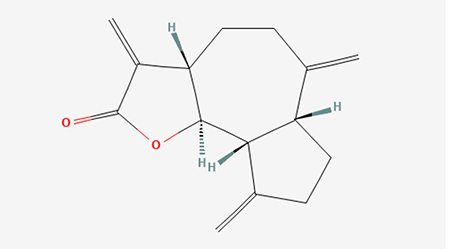
7	(7Z,10Z,13Z)-hexadeca-7,10,13-trienoic acid	C _16_H _26_O _2_	250.38g/mol	13.387	6.49	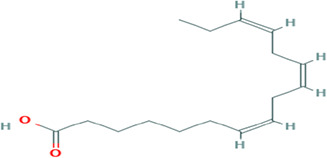
8	Pentadecanoic acid	C _15_H _30_O _2_	242.4g/mol	13.623	7.96	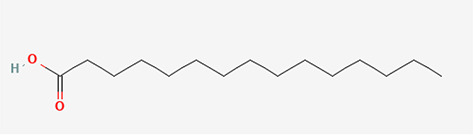
9	Phytol	C _20_H _40_O	296.5g/mol	14.949	4.72	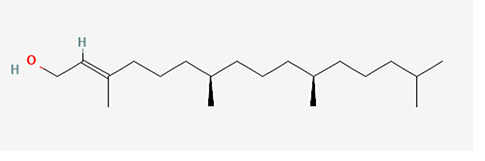
10	(9Z,12Z,15Z)-octadeca-9,12,15-trienoic acid	C _18_H _30_O _2_	278.4g/mol	15.306	13.44	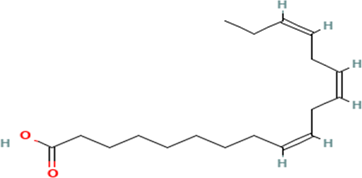
11	Dichloroacetic acid, tridec-2-ynyl ester	C _15_H _24_Cl _2_O _2_	307.3g/mol	15.452	3.00	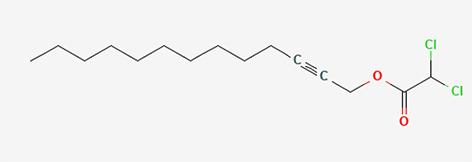
12	2,3-dihydroxypropyl (9Z,12Z,15Z)-octadeca-9,12,15-trienoate	C _21_H _36_O _4_	352.5g/mol	19.817	2.11	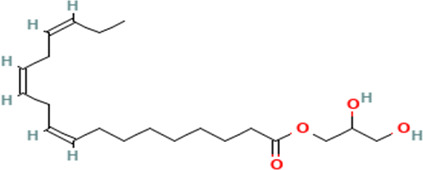
13	Hexatriacontane	C _36_H _74_	507g/mol	21.229	3.46	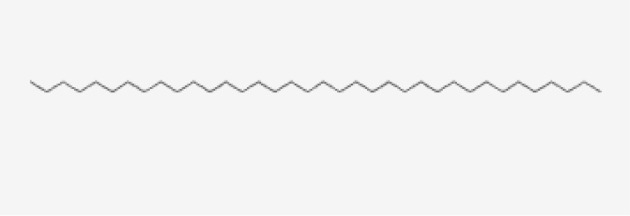
14	gamma.-Sitosterol	C _29_H _50_O	414.7g/mol	25.056	3.25	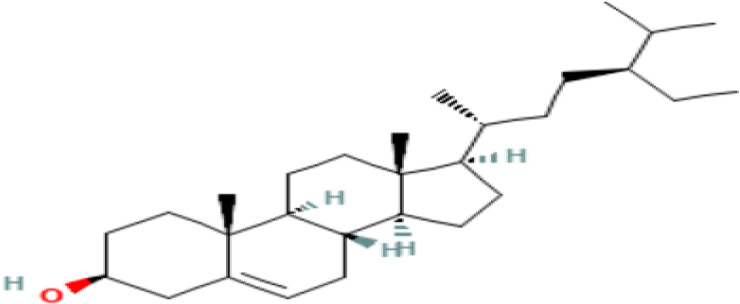

Among the identified compounds are several notable bioactive constituents, including Dimethyl trisulfide, 2-Piperidinemethanamine, 2,7-Dimethyl-1,7-octadien-3-amine, Oxalic acid cyclohexyl propyl ester, 2-Pyrrolidinone, 4H-Pyran-4-one, 2,3-dihydro-3,5-dihydroxy-6-methyl-4H-pyran-4-one, and 2,2-Difluoroethylbenzene. Others include 3,4-Anhydro-d-galactosan, dl-trans-Chrysanthemic acid, Phenol derivatives, Indole, 2-Methoxy-4-vinylphenol, and various pyrrolidine and cyclohexane derivatives.

Further constituents of pharmacological interest include 7,10,13-Hexadecatrienoic acid (Z,Z,Z), 9,12,15-Octadecatrienoic acid methyl ester, phytol, γ-tocopherol, α-tocospiro A and B, stigmasterol, ergost-5-en-3-ol (3β), and γ-sitosterol. Many of these compounds have been previously reported to exhibit antioxidant, anti-inflammatory, antimicrobial, and reproductive health-supportive properties, which may explain the traditional use of collard greens in managing male infertility.

**Figure 3.**
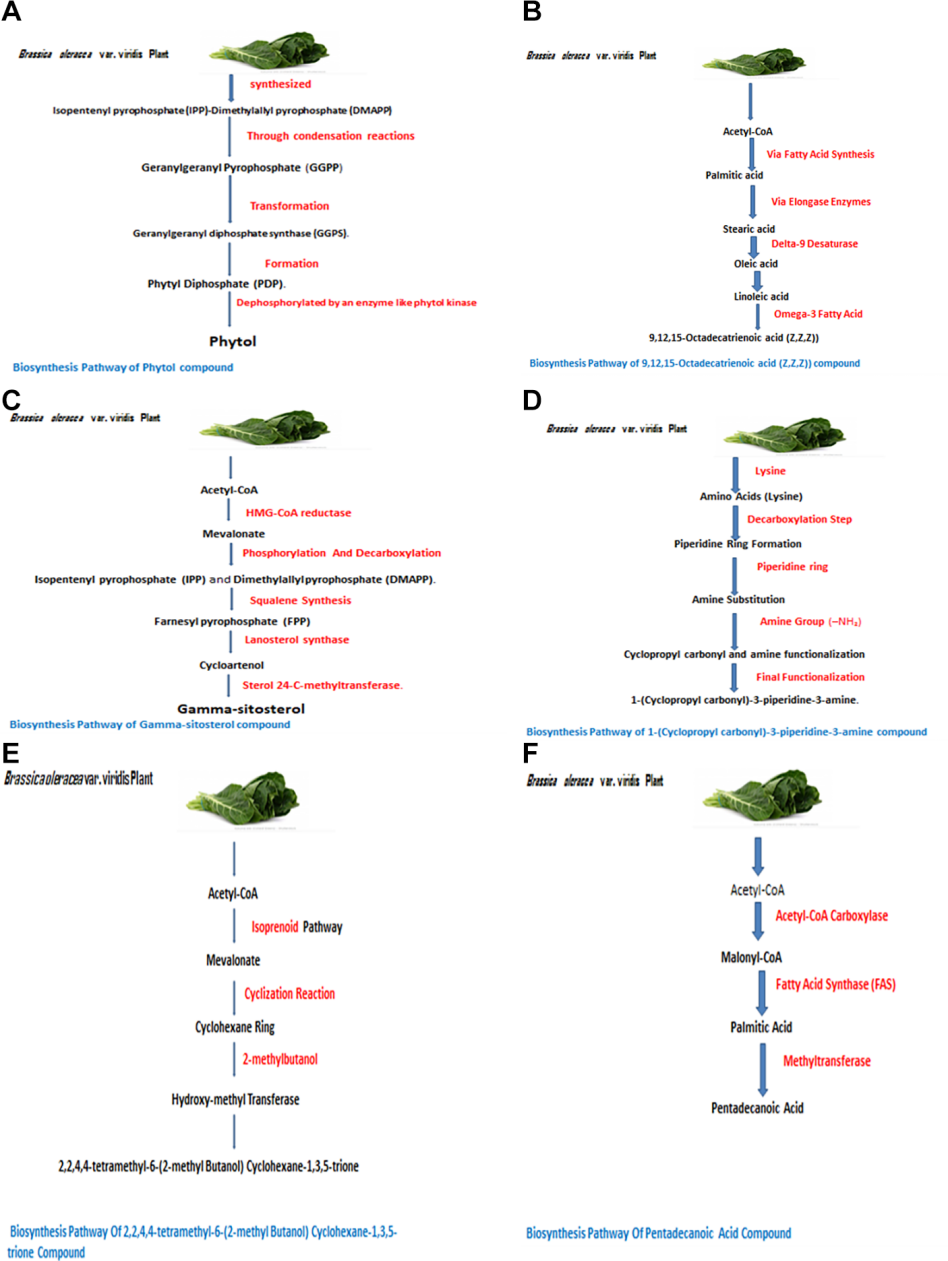
Showing the selected potential compounds and their biosynthesis pathways.

The identified bioactive molecules span multiple chemical classes, including fatty acid esters, phenolics, alkaloids, terpenoids, sterols, and tocopherols, underscoring the pharmacological potential of *Brassica oleracea* var. *viridis.* Selected compounds and their proposed biological pathways relevant to male reproductive health are illustrated in Figure 3.

## Discussion

Phytochemical screening of the ethanol extract from *Brassica oleracea* var. *viridis* (Collard Greens) has revealed a diverse array of phytoconstituents with potential health benefits, particularly in relation to reproductive health, as summarized in Table 4. Among the identified compounds, Phytol, a terpenoid, stands out due to its notable antioxidant properties. Previous studies have demonstrated that Phytol exhibits significant antioxidant activity in vitro, including scavenging hydroxyl radicals and nitric oxide, as well as inhibiting the formation of thiobarbituric acid reactive substances (TBARS). ^13^ These findings are consistent with a systematic review by, ^14^ which linked antioxidants to the restoration of oxidative stress-induced sperm abnormalities. However, it is important to acknowledge the contrasting results presented by, ^15^ who showed that antioxidant supplementation might not enhance semen parameters or DNA integrity in men with male factor infertility. This highlights the complexity of antioxidant supplementation in fertility therapies and suggests that a more nuanced approach may be necessary.

**Table 4.** Showing the selected potential compounds of ethanol extract of *Brassica oleracea* var. viridis (collard greens) predicted for male infertility treatment.

S/N	Compounds	Phytochemical constituents	Potential use for male infertility treatment
1	Phytol	Terpenoids	It supports sperm cell function by enhancing overall cellular health. (Steiner *et al.* 2020)
2	9,12,15-Octadecatrienoic acid, (Z, Z,Z)-, Pentadecanoic acid, 7,10,13-Hexadecatrienoic acid, (Z, Z, Z)-	Fatty Acids	Omega-3 fatty acid, known for improving sperm quality, motility and overall reproductive health.(Safarinejad *et al.* 2012)
3	Phenol, 4-ethenyl-2,6-dimethoxy-	Phenolic	Known for antioxidant properties that may protect sperm from oxidative stress. (Gulcin, 2020)
4	Gamma-Sitosterol	Steroids	Have antioxidant and anti-inflammatory properties, potentially benefiting male fertility. (Reddy *et al.* 2022)

Additionally, the screening identified several omega-3 fatty acids, including 9,12,15-octadecatrienoic acid (Z,Z,Z), pentadecanoic acid, and 7,10,13-hexadecatrienoic acid (Z,Z,Z). These compounds are well-documented for their beneficial effects on sperm quality, motility, and overall reproductive health. A study indicated that omega-3 supplementation could enhance antioxidant activity in human seminal fluid, leading to improvements in sperm count, motility, and morphology. ^16^ A study reported that omega-3 supplementation significantly improved sperm motility, increased docosahexaenoic acid (DHA) levels in seminal plasma, and enhanced total sperm count and cell density. ^17^

The presence of phenolic compounds and flavonoids in the *B. oleracea* var. *viridis* extract also warrants attention, given their potent antioxidant properties. In particular, 4-ethenyl-2,6-dimethoxyphenol has been noted for its potential neuroprotective effects, which may have implications for psychogenic male infertility. ^18^ Furthermore, Gamma-Sitosterol, a plant sterol, exhibits both antioxidant and anti-inflammatory activities, potentially promoting male fertility through modulation of oxidative stress and inflammatory pathways. Research by ^19^ on β-sitosterol further supports its role in inhibiting the growth and spread of prostate cancer cells, which could indirectly enhance reproductive health by preserving prostate function.

## Conclusion

In conclusion, the *Brassica oleracea* var. *viridis* (Collard Greens) ethanol extract contains diverse phytochemicals such as phytol, omega-3 fatty acids, phenolic compounds, flavonoids, and sterols. These phytochemicals play significant potential benefits for enhancing male fertility and reproductive health through antioxidant, anti-inflammatory, and potentially neuroprotective pathways.

### Recommendation

Future research should focus on elucidating the specific mechanisms by which these compounds interact and contribute to male fertility, as well as assessing their clinical efficacy and safety for therapeutic use in reproductive health. These trials should specifically assess the effect of these phytochemicals such as phytol, omega-3 fatty acids, phenols, flavonoids, and sterols on sperm quality, motility, and overall fertility outcomes to establish a clearer understanding of their clinical potential of these phytochemicals in treatment of male infertility.

## Declarations

### Ethics approval

The authors confirm that all guidelines set by the University’s research ethics for plant collection, characterization, and documentation was duly followed. The plant specimen was identified by the Department of Botany, Faculty of Science, Mbarara University of Science and Technology, Uganda. The experimental protocols received approval from the Kampala International University Research Ethics Committee. The plant collection process adhered to local guidelines and does not require further confirmation.

## Data Availability

Open Science Framework: Gas Chromatography-Mass spectrometric (GC-MS) Revealed Bioactive Constituents of *Brassica Oleracea* var. *Viridis* (Collard Greens) Used as Ethnomedicine to Treat Male Infertility in Uganda. ( https://doi.org/10.17605/OSF.IO/QND7X) ^21^ This project contains the following extended data
• ETHANOLIC EXTRACT sukuma wikki file.pdf Data are available under the terms of the Creative Commons Zero “No rights reserved” data waiver (CC0 1.0 Public domain dedication).
