# Supplementary material for: Gas Chromatography-Mass spectrometry (GC-MS) Revealed Bioactive Constituents of Brassica oleracea var. viridis (Collard Greens) Used to Treat Male Infertility in Uganda
Source: F1000Res. 2025 Nov 5;14:525. Originally published 2025 May 28. [Version 3] doi: 10.12688/f1000research.164618.3 (PMC12519067; doi:10.12688/f1000research.164618.3)
Supplement: Peer review history

# Reviewer report: Reviewer response for version 2

**DOI:** 10.5256/f1000research.188560.r417218

**Published:** 2025-10-13

**Authors:** Adeoye-Isijola Morenike

**Affiliations:** 1 Babcock University, Ilishan-Remo, Ogun State, Nigeria

The revisions are are sufficient for me to amend my previous status of "Approved with reservations".

Is the work clearly and accurately presented and does it cite the current literature?

Yes

If applicable, is the statistical analysis and its interpretation appropriate?

I cannot comment. A qualified statistician is required.

Are all the source data underlying the results available to ensure full reproducibility?

Yes

Is the study design appropriate and is the work technically sound?

Yes

Are the conclusions drawn adequately supported by the results?

Yes

Are sufficient details of methods and analysis provided to allow replication by others?

Yes

Reviewer Expertise:

Bioactivity guided medicinal plant research, Phytochemistry

I confirm that I have read this submission and believe that I have an appropriate level of expertise to confirm that it is of an acceptable scientific standard.

# Reviewer report: Reviewer response for version 2

**DOI:** 10.5256/f1000research.188560.r418701

**Published:** 2025-10-01

**Authors:** Amanulla Khan

**Affiliations:** 1 Anjuman Islam Janjira Degree College of Science, Murud Janjira, Raigad, Maharashtra, India

Gas Chromatography-Mass Spectrometry (GC-MS) Revealed Bioactive Constituents of *Brassica oleracea* var. *viridis* (Collard Greens) Used to Treat Male Infertility in Uganda        

  

- Grammar / Style:Minor corrections:
  - “These phytochemicals such as flavonoids, alkaloids…” → *“These phytochemicals, including flavonoids, alkaloids…”*
  - Replace *“as shown in Figure 1”* with a reference to the updated figure.
- Scientific Clarity:
  - Strengths: Clear ethnomedicinal context.
  - Improvements:Cite recent studies (last 5–7 years) on *Brassica oleracea* bioactive compounds and male fertility.Avoid redundancy; streamline phrases like *“These constituents support immune function, bone health, digestion, and oxidative balance”* to maintain focus on reproductive health.         
- Plant Collection & Identification: Detailed and well-documented. Minor grammar correction: *“Fresh leaves (2 kg) … were subsequently transported to the herbarium unit at Mbarara University for proper botanical identification and authentication”* → *“…were transported to the herbarium at Mbarara University for botanical authentication.”*
- Extraction Procedure: ****Clearly mention the ratio (w/v) for ethanol. extraction. Standardize units: *“26 2°C”* → *“26 ± 2 °C”*.Use past tense consistently.
- GC-MS Analysis: Clarify split ratio and sample injection mode. Include column specifications (length, diameter, film thickness) for reproducibility. Tables 2–4: Ensure consistent formatting and capitalization for chemical names. ****Figures: Figure 1: Replace with a high-resolution, original photograph of collard greens collected from the field. Figure 2 (GC-MS chromatogram): Replace with a high-resolution chromatogram; label peaks and retention times clearly. Figure 3 (biosynthetic pathway): Replace with original/redrawn diagrams relevant to the identified compounds; avoid screenshots from other sources.
- Strengths: Detailed plant authentication and voucher specimen deposition. Comprehensive GC-MS analysis. Effective linkage of phytochemicals to male fertility mechanisms.
- Limitations / Recommendations: Include quantitative analysis (concentration of compounds). Discuss potential toxicity or adverse effects. Provide experimental reproducibility details (replicates, GC-MS calibration). Ensure figures and tables are high-quality and original.

The manuscript is scientifically sound and demonstrates a comprehensive GC-MS analysis of *Brassica oleracea* var. viridis. With improvements to figure quality, grammar, formatting, citation updates, and clearer discussion of biological relevance and limitations, this manuscript would meet high standards suitable for indexing.

Is the work clearly and accurately presented and does it cite the current literature?

Yes

If applicable, is the statistical analysis and its interpretation appropriate?

Partly

Are all the source data underlying the results available to ensure full reproducibility?

Partly

Is the study design appropriate and is the work technically sound?

Partly

Are the conclusions drawn adequately supported by the results?

Yes

Are sufficient details of methods and analysis provided to allow replication by others?

Partly

Reviewer Expertise:

Phytochemistry and Medicinal Botany

I confirm that I have read this submission and believe that I have an appropriate level of expertise to confirm that it is of an acceptable scientific standard, however I have significant reservations, as outlined above.

# Response

**Published:** 2025-10-23

**Authors:** Emmanuel Ikuomola

**Affiliations:** Department of Physiology, Kampala International University - Western Campus, Bushenyi, Western Region, Uganda

**Reviewer 3**

 Comments:

- Grammar / Style:Minor corrections:
  - “These phytochemicals such as flavonoids, alkaloids…” → *“These phytochemicals, including flavonoids, alkaloids…”*
  - **Authors’ Response:** Thank you for the correction. The necessary changes have been made in the introductory section of the manuscript.
  - Replace *“as shown in Figure 1”* with a reference to the updated figure.
  - **Authors response**: We thank the reviewer for pointing this out. The phrase has been updated as requested to correctly reference the figure.

 

- Scientific Clarity:
  - Strengths: Clear ethnomedicinal context.
  - **Authors response**: We sincerely thank the reviewer for this positive feedback. We are pleased that the clear presentation of the ethnomedicinal context, which grounds this laboratory investigation in the traditional use of *Brassica oleracea* var. *viridis* for treating male infertility in Uganda, was recognized as a strength of our work.
  - Improvements: Cite recent studies (last 5–7 years) on *Brassica oleracea* bioactive compounds and male fertility.
  - **Authors response: **We thank the reviewer for this valuable suggestion. We have now integrated recent studies (published within the last 5-7 years) into the **manuscript **sections which support the role of *Brassica oleracea* bioactive compounds in promoting male reproductive health. This update strengthens the scientific context of our findings.

 

-
  - Avoid redundancy; streamline phrases like *“These constituents support immune function, bone health, digestion, and oxidative balance”* to maintain focus on reproductive health.   
  - **AUTHORS RESPONSE:** We thank the reviewer for this valuable suggestion. We have streamlined the text as recommended, removing the broad list of general health benefits and refocusing the narrative specifically on the compounds' relevance to male reproductive health. The changes have been made in the **Introduction** section.     

 

- Plant Collection & Identification: Detailed and well-documented. Minor grammar correction: *“Fresh leaves (2 kg) … were subsequently transported to the herbarium unit at Mbarara University for proper botanical identification and authentication”* → *“…were transported to the herbarium at Mbarara University for botanical authentication.”*
- **Authors response**: We thank the reviewer for this positive feedback and for the suggested improvement. The sentence has been revised as recommended to be more concise.

 

- Extraction Procedure: ****Clearly mention the ratio (w/v) for ethanol. extraction. Standardize units: *“26 2°C”* → *“26 ± 2 °C”*.Use past tense consistently.
- **Authors response: **We thank the reviewer for these precise observations. We have clarified the ethanol ratio, corrected the temperature unit, and ensured the consistent use of past tense in the described procedure.
- **Ratio Clarified:** Changed "(1:5 w/v)" to "(at a ratio of 1:5 w/v)" for absolute clarity.
- **Units Standardized:** Corrected "26 2°C" to "26 ± 2 °C" and added a space before "°C" for "40 °C" to ensure consistency.
- **Past Tense & Grammar:** Corrected "was concentrated to dryness reduced pressure" to "was concentrated to dryness under reduced pressure" and "having a constant weight" to "until a constant weight was achieved" for grammatical correctness and consistent use of the past tense.

 

- GC-MS Analysis: Clarify split ratio and sample injection mode. Include column specifications (length, diameter, film thickness) for reproducibility.
- **Authors response: **We thank the reviewer for this critical suggestion to enhance the reproducibility of our methods. We have clarified the split ratio and injection mode and have added the standard column specifications (length, internal diameter, and film thickness) to the GC-MS analysis section.

 

- Tables 2–4: Ensure consistent formatting and capitalization for chemical names. 
- **Authors response:**   We thank the reviewer for highlighting this lack of consistency. We have carefully revised all three tables {2,3 and 4} to ensure chemical names follow standard IUPAC capitalization rules (sentence case for common names, proper capitalization for systematic names) and that formatting is uniform throughout.

 

-  
** **Figures: Figure 1: Replace with a high-resolution, original photograph of collard greens collected from the field. Figure 2 ****(GC-MS chromatogram): Replace with a high-resolution chromatogram; label peaks and retention times clearly. Figure 3 ****(biosynthetic pathway): Replace with original/redrawn diagrams relevant to the identified compounds; avoid screenshots from other sources.
- **Authors response: **We thank the reviewer for these critical recommendations to enhance the quality and originality of our figures. We have taken the following actions:
- **Figure 1:** The original diagram has been replaced with a new, high-resolution, original photograph of *Brassica oleracea* var. *viridis* (collard greens) collected from the field in Bwejuragye, Bushenyi, Uganda, as specified in the methodology.
- **Figure 2:** The GC-MS chromatogram has been replaced with a high-resolution version. Key peaks corresponding to the major bioactive compounds discussed in the manuscript are now clearly labeled with their respective retention times.
- **Figure 3:** The previous pathway diagram has been removed. It has been replaced with an original, self-created schematic that illustrates the proposed mechanistic pathways.

 

- Strengths: Detailed plant authentication and voucher specimen deposition. Comprehensive GC-MS analysis. Effective linkage of phytochemicals to male fertility mechanisms.
- **Authors response:** We sincerely thank the reviewer for their positive assessment and for recognizing the strengths of our work, particularly the rigorous plant authentication, comprehensive phytochemical profiling, and the functional interpretation of the results in the context of male fertility.

 

- Limitations / Recommendations: Include quantitative analysis (concentration of compounds). Discuss potential toxicity or adverse effects. Provide experimental reproducibility details (replicates, GC-MS calibration). Ensure figures and tables are high-quality and original.
- **Authors response: **We thank the reviewer for these critical and constructive points. We have addressed them by adding a dedicated "Limitations and Future Perspectives" section to the manuscript. The key additions are summarized below.

**Key additions **

1. **Added a New Section:** A "Limitations and Future Perspectives" section has been inserted before the conclusion.
2. **Addressed Quantification:** Explicitly stated the qualitative/semi-quantitative nature of the study and recommended quantitative analysis for future work.
3. **Addressed Toxicity:** Acknowledged the lack of toxicity data and recommended future toxicological studies.
4. **Addressed Reproducibility:** Specified that the analysis was done in triplicate and mentioned instrument calibration.
5. **Confirmed Figure Quality:** Reiterated that all figures have been replaced with high-quality, original versions as per the reviewer's previous comment.

# Reviewer report: Reviewer response for version 1

**DOI:** 10.5256/f1000research.181158.r390229

**Published:** 2025-09-05

**Authors:** Adeoye-Isijola Morenike

**Affiliations:** 1 Babcock University, Ilishan-Remo, Ogun State, Nigeria

COMMENTS:  

1. The topic needs to be revised if possible and, should read: " Gas Chromatography - Mass spectrometry.......(not "spectrometric"). 
2. The revision of the topic is suggested because ethnomidicine is the basis for suggesting that the *Brassica oleracea* var. Viridis is used to treat male infertility in Uganda and no previous scientific evidence is referenced or alluded to. Also, reports of the ethnobotanical survey should be included, if available. 
3. Please note that:

- Scientific names are always italicized 
- The genus is always capitalized 
- The species is never capitalized     

4. The method in the abstract says the "point of this study was to find out...." 

- What bioactive compounds might be in the ethanol extract of collard green leaves 
- how well they work as antioxidants. 
- .....may be responsible for their traditional use in treating male fertility 

Note that every objective (as shown above) must have clearly defined materials and methods but the only materials and methods seen is that of the GC-MS which addresses only the first objective which is to "find out what bioactive compounds might be in the ethanol extract of collard green leaves" 

 The second objective mentioned, which was to "find out how well they work as antioxidants" was not attended to by any materials and method carrying out the antioxidant capacity assays such as  DPPH, ABTS, FRAP, NO or any other of the tests for radical scavenging activity, lipid peroxidation, reducing power; TEAC and TRAP are assays with mechanisms specifically for antioxidant activity

 5. In the introduction, the statement below, must be punctuated with an appropriate reference compulsorily because the later part of the sentence is the crutch on which the justification for the research work is resting on:

 "Additionally preliminary evidence suggests potential anti-hyperglycemic and fertility-enhancing effects, reinforcing its traditional use in managing male infertility" (reference not included)

Is the work clearly and accurately presented and does it cite the current literature?

Yes

If applicable, is the statistical analysis and its interpretation appropriate?

I cannot comment. A qualified statistician is required.

Are all the source data underlying the results available to ensure full reproducibility?

Yes

Is the study design appropriate and is the work technically sound?

Yes

Are the conclusions drawn adequately supported by the results?

Yes

Are sufficient details of methods and analysis provided to allow replication by others?

Yes

Reviewer Expertise:

Bioactivity guided medicinal plant research, Phytochemistry

I confirm that I have read this submission and believe that I have an appropriate level of expertise to confirm that it is of an acceptable scientific standard, however I have significant reservations, as outlined above.

# Response

**Published:** 2025-09-21

**Authors:** Emmanuel Ikuomola

**Affiliations:** Department of Physiology, Kampala International University - Western Campus, Bushenyi, Western Region, Uganda

“We sincerely thank the reviewer for their constructive feedback and valuable recommendations. The authors’ detailed responses to the comments are provided below.”

 Reviewer Comments:  

1. The topic needs to be revised if possible and, should read: " Gas Chromatography - Mass spectrometry.......(not "spectrometric"). 

Author’s **Response**: The topic has been revised to read “Gas chromatography-Mass spectrometry” as suggested

 2. The revision of the topic is suggested because ethnomedicine is the basis for suggesting that the *Brassica oleracea* var. Viridis is used to treat male infertility in Uganda and no previous scientific evidence is referenced or alluded to. Also, reports of the ethnobotanical survey should be included, if available.  Author’s **Response**: The topic has been revised the word ethnomedicine has been deleted to read “Gas Chromatography-Mass spectrometry (GC-MS) Revealed Bioactive Constituents of *Brassica Oleracea *var. *Viridis* (Collard Greens) Used to Treat Male Infertility in Uganda. However, the report of the ethnobotanical survey is not available.

 3. Please note that:

- Scientific names are always italicized 
- The genus is always capitalized 
- The species is never capitalized   

Author’s **Response:** The botanical name has been corrected to show the genus and species in small letters.  

 4. The method in the abstract says the "point of this study was to find out...." 

- What bioactive compounds might be in the ethanol extract of collard green leaves 
- how well they work as antioxidants. 
- .....may be responsible for their traditional use in treating male fertility 

Note that every objective (as shown above) must have clearly defined materials and methods but the only materials and methods seen is that of the GC-MS which addresses only the first objective which is to "find out what bioactive compounds might be in the ethanol extract of collard green leaves" 

 The second objective mentioned, which was to "find out how well they work as antioxidants" was not attended to by any materials and method carrying out the antioxidant capacity assays such as  DPPH, ABTS, FRAP, NO or any other of the tests for radical scavenging activity, lipid peroxidation, reducing power; TEAC and TRAP are assays with mechanisms specifically for antioxidant activity.

 Author’s **Response**: The objective has been modified to focus on only the GC-MS. The sentence now reads “The aim of this study was to identify bioactive compounds in the ethanol extract of collard green leaves that has traditional use in treating male infertility in Uganda”.

 5. In the introduction, the statement below, must be punctuated with an appropriate reference compulsorily because the later part of the sentence is the crutch on which the justification for the research work is resting on:

 "Additionally preliminary evidence suggests potential anti-hyperglycemic and fertility-enhancing effects, reinforcing its traditional use in managing male infertility" (reference not included).

 Author’s **Response**: The supporting references have been included in the introduction as suggested. The reference numbers are 6 and 7.

# Reviewer report: Reviewer response for version 1

**DOI:** 10.5256/f1000research.181158.r388513

**Published:** 2025-06-25

**Authors:** Faten Hameed Thamer

**Affiliations:** 1 Sana'a University, Sana'a, Yemen

**General comments**

- The abstract is well-structured but can benefit from clearer language, more detail about methods, and precise emphasis on the significance of findings.
- Clarify the methodology details, especially regarding extraction and GC-MS parameters.
- Highlight the potential health implications of identified compounds more explicitly.
- Consider adding quantitative data if available, such as relative abundances of key compounds.
- Ensure consistency in terminology, e.g., "bioactive compounds" vs. "phytochemicals."
- Improve readability with more formal academic language, especially in the conclusion.

**1. Introduction to sample collection:**

- Clarify why the specific location was chosen, if relevant.
- Correctly cite the method if it is referenced and complete the citation.

**2. Botanical identification:**

- Specify the authority or botanist who confirmed the identification for scientific rigor (e.g., "identified and authenticated by Dr. XYZ using standard taxonomic keys").

**3. Sample preparation details:**

- Mention how many leaves were collected or the approximate weight of the fresh leaves before drying.
- Specify the drying conditions (e.g., "dried at ambient temperature (~25°C) in the shade" for accuracy).

**4. Extraction process:**

- Complete the citation for the methodology or clarify if it’s a modified version (e.g., "based on the method described by [Author], [Year]").
- Mention the solvent volume in a consistent manner ("5.5 liters of 99% ethanol," which is clear).

**5. Maceration process:**

- Indicate the container type used and mention if the mixture was shaken periodically during the maceration.
- Clarify whether the mixture was kept in the dark to prevent light degradation.

**6. Filtration and concentration:**

- Specify the conditions of the rotary evaporator, such as temperature (e.g., "at 40°C") and pressure.
- Mention how the dryness was confirmed (e.g., no further solvent smell or weight stabilization).

**7. Storage:**

- Clarify storage conditions (e.g., temperature, container type).

Is the work clearly and accurately presented and does it cite the current literature?

Yes

If applicable, is the statistical analysis and its interpretation appropriate?

I cannot comment. A qualified statistician is required.

Are all the source data underlying the results available to ensure full reproducibility?

Yes

Is the study design appropriate and is the work technically sound?

Yes

Are the conclusions drawn adequately supported by the results?

Yes

Are sufficient details of methods and analysis provided to allow replication by others?

Partly

Reviewer Expertise:

bioactivity analysis, phytochemistry

I confirm that I have read this submission and believe that I have an appropriate level of expertise to confirm that it is of an acceptable scientific standard, however I have significant reservations, as outlined above.

# Response

**Published:** 2025-09-21

**Authors:** Emmanuel Ikuomola

**Affiliations:** Department of Physiology, Kampala International University - Western Campus, Bushenyi, Western Region, Uganda

“We sincerely thank the reviewer for their constructive feedback and valuable recommendations. The authors’ detailed responses to the comments are provided below.”

**General reviewer comments**

 The abstract is well-structured but can benefit from clearer language, more detail about methods, and precise emphasis on the significance of findings.

 Clarify the methodology details, especially regarding extraction and GC-MS parameters.

 Author’s **Response**: The abstract has been modified to contain method of extraction and conclusion emphasized the significance of the phytochemicals identified in the extract.

 Author’s **Response:** The method of extraction has been clarified by including the temperature, volume of solvent used and the storage of the extract obtained in page 4.

 Highlight the potential health implications of identified compounds more explicitly.

 Consider adding quantitative data if available, such as relative abundances of key compounds.

 Ensure consistency in terminology, e.g., "bioactive compounds" vs. "phytochemicals."

 Author’s **Response:** The quantitative data is not available however table three shows the retention time and area under curve that represents a little bit of abundance of the compounds.

 The terminology "bioactive compounds" has been replaced with “phytochemicals” as suggested for consistency.

 Improve readability with more formal academic language, especially in the conclusion.

 1. Introduction to sample collection:

 Clarify why the specific location was chosen, if relevant.

 Author’s **Response**: This specific location was chosen since it has more farmland where the plant is cultivated. The GPS location has been included in the text for clarity.

 Correctly cite the method if it is referenced and complete the citation.

 Author’s **Response:** There is no referenced method of picking the plant. It was just by plucking the leaves from the plant.

 2. Botanical identification:

 Specify the authority or botanist who confirmed the identification for scientific rigor (e.g., "identified and authenticated by Dr. XYZ using standard taxonomic keys").

 Author’s **Response:** The plant was identified and authenticated by Dr. Eunice Olet of the Department of Botany, Faculty of Biological Sciences, Mbarara University of Science and Technology. A voucher specimen was deposited in the university's herbarium under the reference number IOE-24-001.

 3. Sample preparation details:

 Mention how many leaves were collected or the approximate weight of the fresh leaves before drying.

 Author’s **Response:** The quantity of the fresh leaves has been mentioned to be 2 kg in page 4.

 Specify the drying conditions (e.g., "dried at ambient temperature (~25°C) in the shade" for accuracy).

 Author’s **Response:** The drying condition has been specified as shed dried at ambient temperature of 26℃.

 4. Extraction process:

 Complete the citation for the methodology or clarify if it’s a modified version (e.g., "based on the method described by [Author], [Year]").

 Author’s **Response**: The sentence has been completed to read “The extraction process was performed using a modified version of the method described by Abdulrahman et al. ^10^” in page 4.

 Mention the solvent volume in a consistent manner ("5.5 liters of 99% ethanol," which is clear).

 Author’s **Response**: The solvent volume has been modified to read “A total of 800 g of the powdered leaves was cold macerated in 5 liter of 99% ethanol (1:5 w/v).”

 5. Maceration process:

 Indicate the container type used and mention if the mixture was shaken periodically during the maceration.

 Author’s **Response:** The container has been specified as “sterile glass container”. The mixture was shaken periodically during the maceration process. This has been stated in page 4.

 Clarify whether the mixture was kept in the dark to prevent light degradation.

 Author’s **Response**: The mixture was kept in the dark as stated in page 4.

 6. Filtration and concentration:

 Specify the conditions of the rotary evaporator, such as temperature (e.g., "at 40°C") and pressure.

 Author’s **Response:** The temperature is stated as below 40°C and under reduced pressure. This is stated in the relevant section on page 4.

 Mention how the dryness was confirmed (e.g., no further solvent smell or weight stabilization).

 Author’s **Response:** The filtrate was evaporated to dryness having obtained a constant weight as stated in page 4.

 7. Storage:

 Clarify storage conditions (e.g., temperature, container type).

 Author’s **Response:** The storage condition of the extract has been mentioned as follows in page 4. “The dried crude extracts were stored at 4°C in an amber glass bottle that was tightly sealed until further analysis”.
